# Intermediate complex morphophysiological dormancy in seeds of *Aconitum barbatum* (Ranunculaceae)

**DOI:** 10.1186/s12870-023-04357-x

**Published:** 2023-07-05

**Authors:** Lei Zhang, Chaohan Xu, Huina Liu, Qingqing Wu, Jun Tao, Keliang Zhang

**Affiliations:** College of Horticulture and Landscape Architecture, Yangzhou University, Yangzhou, 225009 China

**Keywords:** *Aconitum barbatum*, Morphophysiological dormancy, Cold stratification, Seed dormancy and germination, Underdeveloped embryos, Ranunculaceae

## Abstract

**Background:**

Seed dormancy and germination are key components of plant regeneration strategies. *Aconitum barbatum* is a plant commonly found in northeast China. Although it has potential for use in gardening and landscaping, its seed dormancy and regeneration strategy, which adapt to its natural habitat, are not well understood. Our aim was to identify conditions for breaking *A. barbatum* seed dormancy and determine its dormancy type. Embryo growth and germination were determined by collecting seeds over time in the field. Laboratory experiments that control light, temperature, and stratification period were conducted to assess dormancy breaking and germination, and GA_3_ was used to identify dormancy type.

**Results:**

Seeds of *A. barbatum* have undeveloped embryos with physiological dormancy at maturity in autumn. The embryo-to-seed length ratio increases from 0.33 to 0.78 before the emergence of the radical. Under natural environmental conditions, embryo development begins in early winter. Laboratory experiments have shown that long-term incubation under 4 °C (cold stratification) promotes embryo development and seed dormancy break. With an extension of cold stratification, an increase in germination percentages was observed when seeds were transferred from 4 °C to warmer temperatures. Seeds exposed to light during incubation show a higher germination percentage than those kept in the dark. Seed germination can also be enhanced by a 100 mg/L GA_3_ concentration.

**Conclusions:**

Seeds of *A. barbatum* display intermediate complex morphophysiological dormancy at maturity. In addition to the underdeveloped embryo, there are also physiological barriers that prevent the embryo from germinating. Dormancy breaking of *A. barbatum* seeds can be achieved by natural winter cold stratification, allowing seeds to germinate and sprout seedlings at the beginning of the following growing season. Our findings provide valuable insights into the seed dormancy and regeneration strategy of *A. barbatum*, which could facilitate its effective utilization in gardening and landscaping.

## Background

The periods of seed germination and seedling establishment are widely regarded as crucial stages for plants, and they are believed to have a significant impact on the composition of plant communities and their distribution across different locations [1–3]. To ensure successful germination at the appropriate time and location, plants have developed a range of strategies [4–6]. Among these, the presence of underdeveloped embryos in their seeds is a common approach for plant species across multiple families [6, 7]. These seeds may be classified into morphologically dormant (MD) by Nikolaeva [8], which implies that the embryo has to grow to a certain amount prior to seed germination. Many temperate species with MD have another physiological mechanism that induces seed dormancy, also referred to as morphophysiologically dormant (MPD) [9–11]. Nine MPD levels are determined based on the following factors: (1) temperature needed for breaking seed dormancy; (2) temperature necessary for embryo development, as well as (3) ability to replace cold/warm stratification by GA_3_ to break dormancy [7, 12].

The Ranunculaceae (buttercup family) is a flowering plant family that includes approximately 2,000 species in 43 genera [13]. Some species, like *Aconitum napellus*, *Clematis recta*, and *Hydrastis canadensis*, have medicinal properties and are used in homeopathy [14–16]. Additionally, several genera in this family, including *Aconitum*, *Clematis*, *Consolida*, *Delphinium*, *Helleborus*, and *Trollius*, are popular for their unique flower morphologies and are cultivated for ornamental horticulture [17]. Because the ripe embryo of the Ranunculaceae is typically tiny, this family may be in morphological or morphophysiological dormancy. For instance, seeds of *Pulsatilla slavica* have been suggested with MD [18]; seeds of *Adonis vernalis* [19], *Anemone coronariab* [20], *Aquilegia oxysepala* [21] show nondeep simple MPD; Seeds of *Aconitella decipiens* [19] were reported to have intermediate simple morphophysiological dormancy; seeds of *Aconitum altaicum* [19] show deep simple MPD; seeds of *Actaea pachypoda* [7], and *A. racemosa* [22, 23] were reported to show deep simple epicotyl MPD, while seeds of *Anemone ranunculoides* [24] were reported to show nondeep simple epicotyl morphophysiological dormancy.

*Aconitum barbatum* is a member of the *Aconitum* genus, which belongs to the Ranunculaceae family. This plant can be found growing on grassy slopes, in forests, and on mountains and hills, with altitudes ranging from 400 to 2700 m in the northern regions of China such as Heilongjiang, Jilin, Shanxi, and Shaanxi, and it is also present in Russia [25]. This species has been widely cultivated as a horticultural plant in the northern parts of China and thrives in wet and well-drained loamy soil. Additionally, although it is incredibly potent and carries a high risk of overdose due to its toxic effects, it has been used in folk medicine with necessary caution for treating various ailments such as asthma, diabetes mellitus (DM), nose infections, ear infections, paralysis, leprosy, and arthritis [26].

In recent years, *A. barbatum* has faced a growing threat from increasing market demand as well as extensive human activities that have caused unprecedented disturbance to its natural habitat. Furthermore, germination of *A. barbatum* seeds is difficult, resulting in high costs for propagation and a significant amount of seed wastage. However, despite these challenges, there has been a lack of research into the dormancy characteristics of *A. barbatum* seeds and their regenerative strategies, which are specifically adapted to their natural habitat. Therefore, much remains to be learned about the mechanisms underlying seed dormancy and how this species can successfully thrive in its environment. Investigating these aspects can provide insights into effective methods for breaking seed dormancy, optimizing propagation and cultivation, and conserving the species. Ultimately, such research can help to promote sustainable use of *A. barbatum* and ensure its continued survival in the face of ongoing anthropogenic pressures.

We therefore focused on investigating seed dormancy type and level in *A. barbatum*, as well as its germination prerequisites. We specifically investigated: (1) the effects of light and temperature on seed germination; (2) the effects of warm/cold stratification on embryo growth; (3) the loss of seed dormancy through burial experiments; and (4) the effects of GA_3_ on seed germination, which could help identify the seed dormancy types.

## Results

### Seed mass and water content

The seeds of *A. barbatum* are triangular and pyramidal in morphology, with small, transverse membrane lamellae. The length of the seeds is 3.01 ± 0.05 mm, the width is 2.10 ± 0.01 mm, and the thickness is 1.44 ± 0.02 mm. The obtained thousand-grain weight and seed water content were 1.6077 ± 0.0059 g and 9.62 ± 0.13% respectively. In the observation, there is no obvious difference among the seeds, and they all belong to small seeds.

### Imbibition tests

**Fig. 1:**
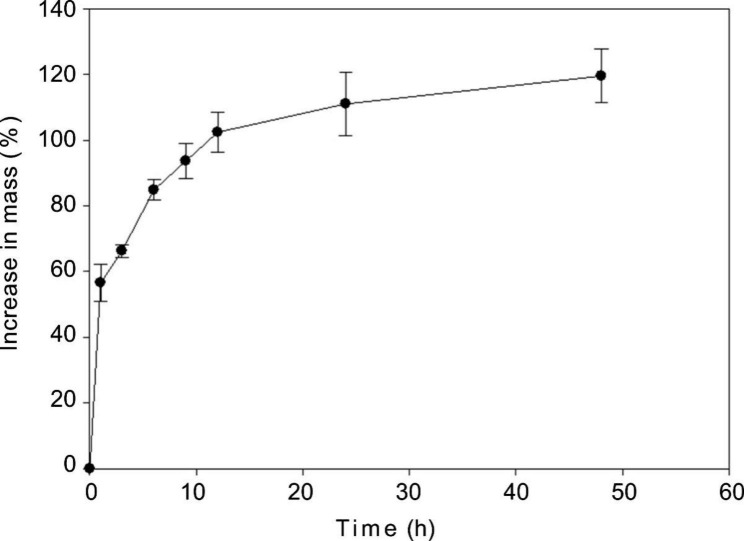
Seed imbibition curves (mean ± s.e.) of *Aconitum barbatum*

As can be seen from the Fig. 1, seeds of *A. barbatum* were able to absorb water. Speed of water absorption more quickly in the beginning of the water absorption. After 1 and 12 h, 56.68 ± 5.60% and 111.06 ± 9.44% increases in seed mass (Fig. 1).

### Germination and embryo length of fresh seeds

**Fig. 2:**
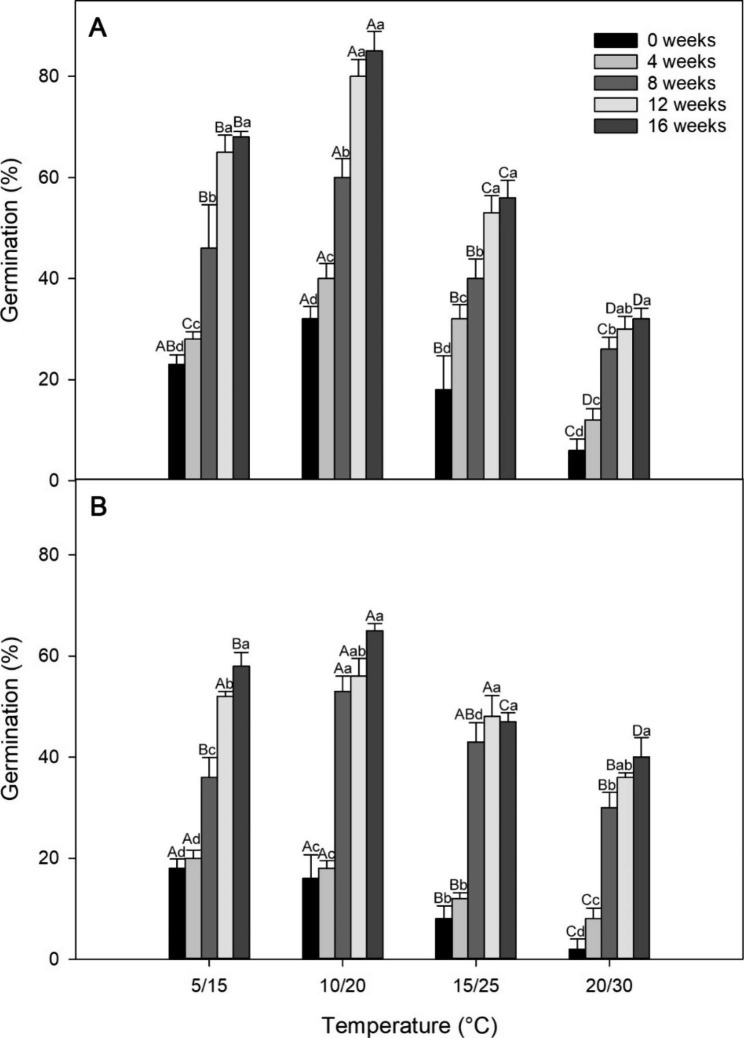
Germination percentage (mean ± s.e.) of *Aconitum barbatum* seeds after various weeks of cold stratification at 4 °C in light **(A)** and dark **(B)**. Different capital and lowercase letters show significant differences between different incubation temperatures during the same cold stratification intervals and different cold stratification periods during the same incubation temperature (P < 0.05), respectively

The embryo:seed (E/S) ratio in fresh seeds was 0.33 ± 0.01. After 1 month of incubation under 5/15, 10/20, 15/25 and 20/30°C, 23%, 32%, 18%, and 6% seeds germinated in light, whereas 18%, 16%, 8% and 2% in dark (Fig. 2). A two-way ANOVA indicated that light had a significant effect on germination (F = 5.334, p < 0.05), but temperature (F = 0.493, p = 0.691) and the interaction effects of temperature and light (F = 0.144, p = 0.933) did not.

### Effects of cold/warm stratification on germination of *A. barbatum* seeds

A three-way ANOVA indicates that light (F = 12.67, p < 0.05), cold stratification period (F = 67.89, p < 0.05), and incubation temperature (F = 26.58, p < 0.05), as well as all their 2 or 3-way interactions (p < 0.05), had a significant effect on the germination of *A. barbatum* seeds. Specifically, the proportion of germination elevated with extension of cold stratification. Additionally, seeds under light incubation showed an increased germination percentage compared with those under dark incubation. Seeds under incubation at 15/25 and 20/30°C displayed a decreased germination percentage compared with those under 5/15 and 10/20°C incubation (Fig. 2). Following incubation at 5/15, 10/20, 15/25, and 20/30°C for 8 weeks, 46%, 60%, 40%, and 26% seeds germinated in light, and 36%, 53%, 42%, and 30% seeds germinated in dark, respectively. After 16 weeks of incubation, 68%, 85%, 56%, and 32% seeds germinated in light, and 58%, 65%, 47%, and 40% seeds germinated in dark, respectively. On the contrary, following warm stratification for a 16-week period, no seed germination was observed following 5/15, 10/20, 15/25, and 20/30°C and light or darkness incubation for a 30-day period.

### Effects of temperature on embryo growth of *A. barbatum* seeds

**Fig. 3:**
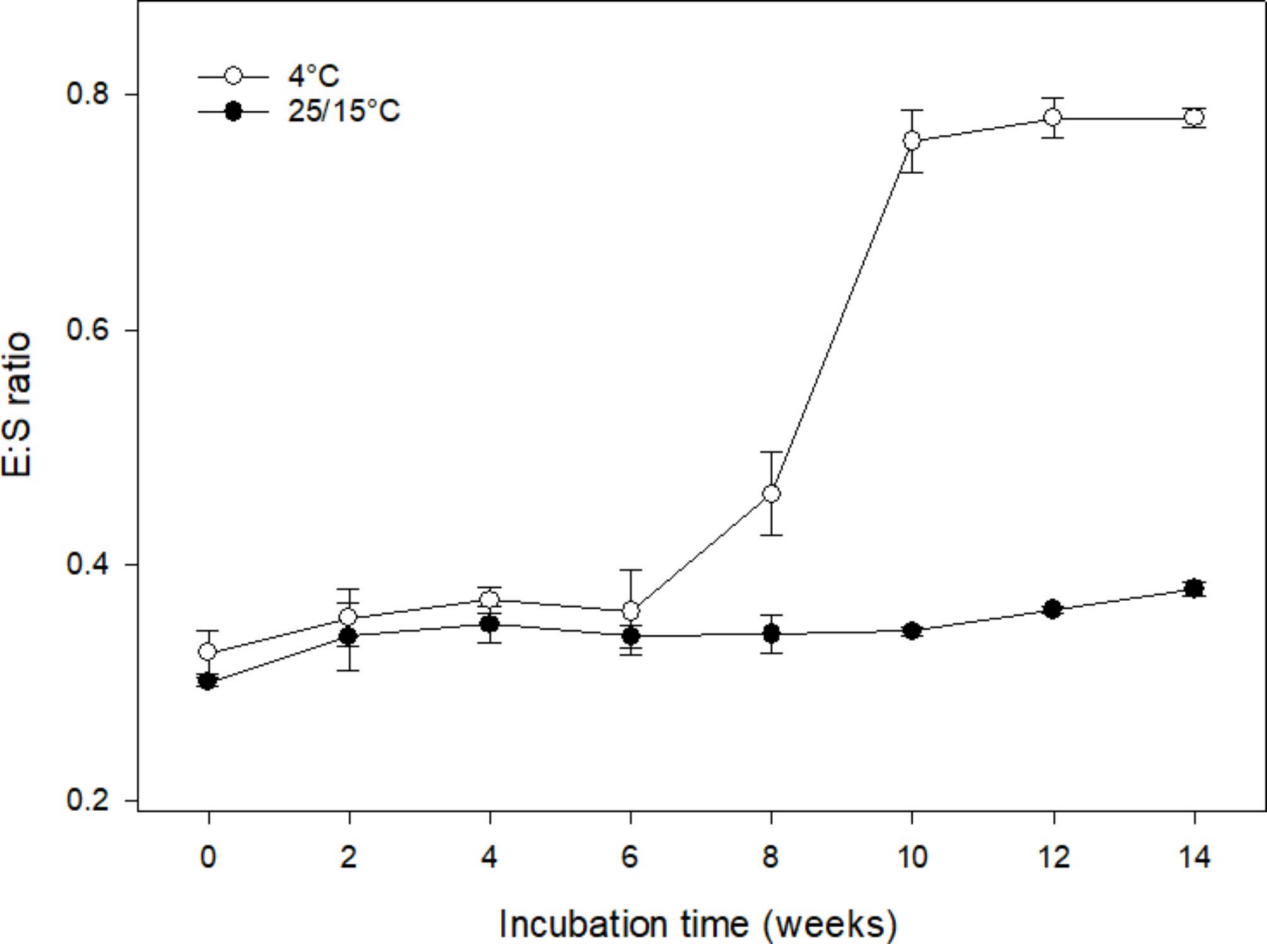
Effects of incubation temperature on embryo: seed length ratio (mean ± s.e.) of *Aconitum barbatum* seeds

A two-way ANOVA revealed that incubation period (F = 15.418, P < 0.05), incubation temperature (F = 7.633, P < 0.05), as well as their interaction (F = 2.833, P < 0.05) significantly affected the E/S ratio of *A. barbatum* seeds. During the 0–6 week incubation period, seed E/S ratios under warm and cold stratification increased slowly (Fig. 3). After a 6-week cold stratification at 4 °C, the embryos grew rapidly, and the E/S ratio increased by 2.6-fold in 4 weeks, reaching to 0.78. In contrast, the E/S ratio remained almost unchanged during a 14-week incubation period at 15/25°C.

### Effects of GA_3_ on germination of *A. barbatum* seeds

**Fig. 4:**
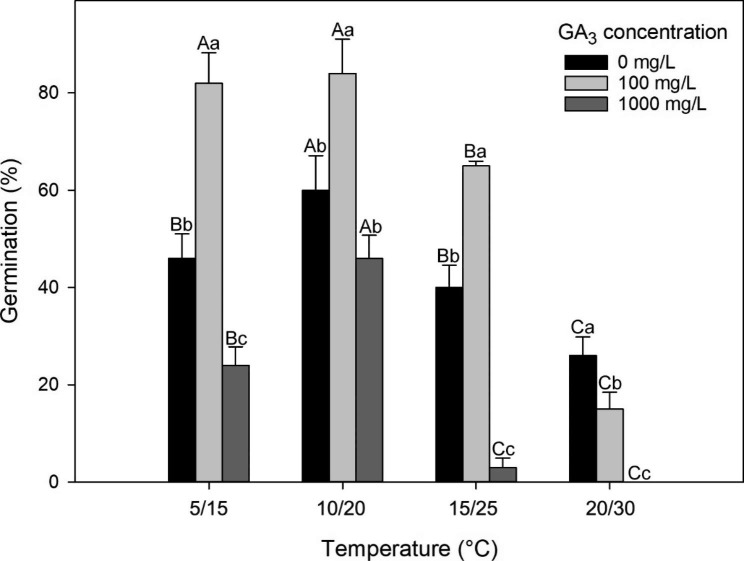
Effects of GA_3_ concentration and incubation temperature on germination percentages (mean ± s.e.) of *Aconitum barbatum* seeds. Different capital and lowercase letters show significant differences between different incubation temperatures under same GA_3_ concentration and different GA_3_ concentration under the same incubation temperature (P < 0.05), respectively

A two-way ANOVA revealed that GA_3_ concentration (F = 135.236, P < 0.05), incubation temperature (F = 13.495, P < 0.001), as well as their interaction (F = 6.726, P < 0.05) significantly affected the germination of *A. barbatum* seeds (Fig. 4). Specifically, a 100 mg/L GA_3_ enhanced germination of *A. barbatum* seeds, with 82%, 84%, 65%, and 15% germinating after 12 weeks of incubation at 5/15, 10/20, 15/25, and 20/30℃, respectively. However, a concentration of 1000 mg/L GA_3_ hindered seed germination, with the seed germination percentages of 24%, 46%, 3% and 0% following 12-week incubation.

### Germination phenology and embryo growth

**Fig. 5:**
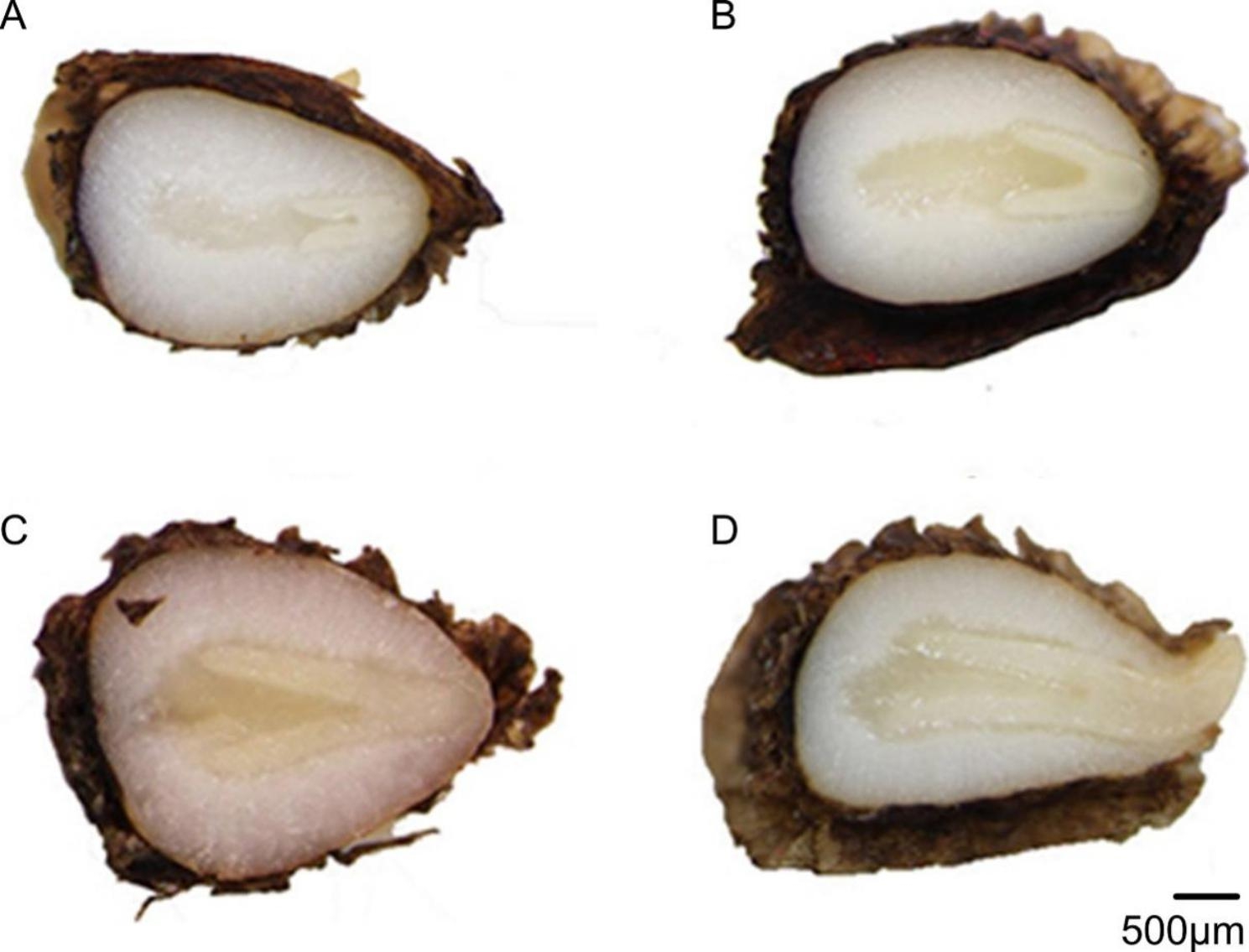
Longitudinal sections of *Aconitum barbatum* seeds buried in soil at a depth of 2 cm in 2018–2019. Fresh seeds having rudimentary embryos (**A**), and growing embryos when seeds exhumed on 1 December 2019 (**B**), 15 January 2019 (**C**), and 1 February 2019 (**D**)

**Fig. 6:**
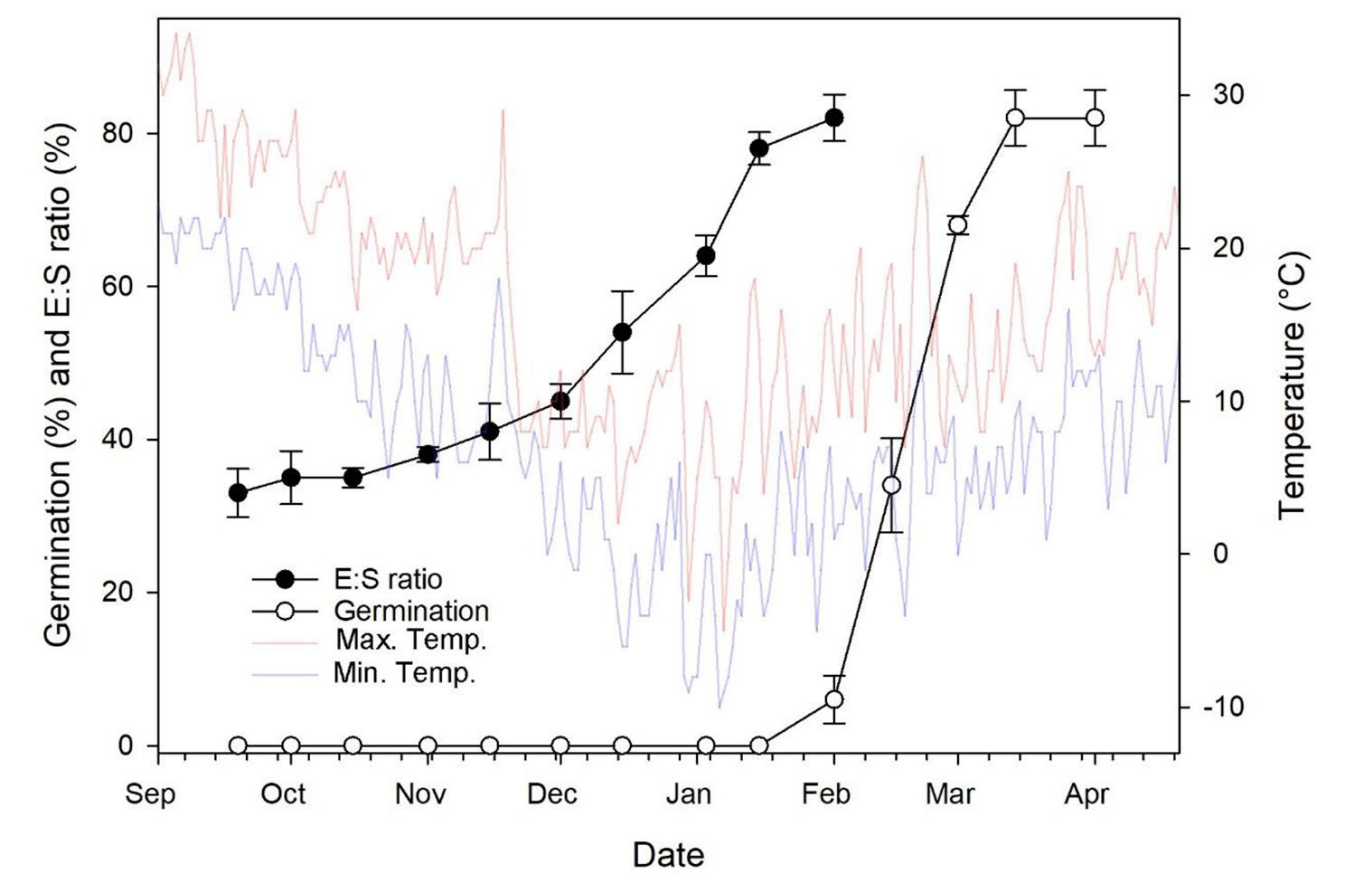
Mean daily maximum and minimum air temperatures, embryo growth phenology, and cumulative germination of *Aconitum barbatum* seeds buried at a depth of 2 cm in soil were recorded from September 2018 to April 2019. Error bars are ± se

A significant development in the *A. barbatum* seed embryo was observed at mid-November (Fig. 5), with the daily maximal and minimal temperatures being 16.89 and 8.96 °C, respectively. Embryo length gradually increased during the winter season (Fig. 6). In contrast, seed germination did not occur until the beginning of February, with daily maximal and minimal temperatures of 10.02 and 4.87 °C, respectively. The highest seed germination percentage was observed on March 14th, with daily maximal and minimal temperatures of 16.92 and 6.35 °C, respectively. At this time, more than 80% of the seeds had germinated (Fig. 6).

## Discussion

Freshly ripened *A. barbatum* seeds were subjected to incubation under alternating temperature conditions in the light and dark, with 5/15, 10/20, 15/25, and 20/30°C, resulting in 23%, 32%, 18%, and 6% of seeds germinating in the light, implying that most seeds were in a dormant state. Unlike species that feature combinational (physical plus physiological) or physical dormancy [7], seeds of *A. barbatum* could imbibe water without requiring scarification pretreatment (Fig. 1). Indeed, either combinational or physical dormancy had not been reported in seeds of *Aconitum* and those of other Ranunculaceae species [7, 21]. In addition, the E/S ratio of fresh seeds was determined to be 0.33 ± 0.01, however, after cold stratification for a 10-week period (Fig. 3), the E/S ratio increased 2.6-fold. According to Baskin and Baskin [7], seeds had MD if embryo growth and seed germination occurred within 30 days at the proper temperatures without any dormancy breaking treatment; seeds are considered to have MPD when underdeveloped embryos were observed in seeds that required dormancy breaking treatment, such as warm or cold stratification [7]. Given all seeds of *A. barbatum* had underdeveloped embryos and require growth prior to germination, we conclude that most seeds of *A. barbatum* had morphophysiological dormancy, while a small portion of seeds exhibit morphological dormancy.

Understanding the conditions necessary for embryo development is essential for categorizing the level of MPD. There are nine levels of MPD that have been established, which are further divided into two types: simple and complex, based on temperatures required for embryo development. In simple MPD, embryo development can be observed under warm stratification (≥ 15 °C), while in complex MPD, embryo development can be observed under cold stratification (about 0 to 10 °C) [7, 12, 28]. In our study, we observed embryo growth of *A. barbatum* occurred at cold temperature (Fig. 3). Furthermore, the germination percentage of *A. barbatum* seeds increased with increasing cold stratification periods (Fig. 2), suggesting that a longer time under low temperature is needed for breaking physiological dormancy in many seeds. In the field, embryo growth occurs quickly after freshly matured seeds are exposed to cold stratification (Fig. 6). All of which suggests that low temperatures are necessary for embryo development. Consequently, *A. barbatum* seeds are suggested with the complex type MPD.

Additionally, the PD component of MPD is also divided into nondeep, intermediate, or deep type, based on how the seed reacts to GA_3_ pretreatment [7]. Seeds have non-deep or intermediate PD when GA_3_ substitutes the necessity for cold or warm temperature to achieve dormancy breaking. Seeds have deep PD if GA_3_ cannot replace the need for warm or cold temperature [7]. Seeds with nondeep PD can be broken by either cold or warm stratification, whereas seeds with intermediate PD can only be broken by cold stratification. Furthermore, the cold stratification period required to break intermediate PD, typically lasting 2–3 months, is longer than that required to break nondeep PD, which is usually ranges from 5 to 60 days for seeds with nondeep PD that require cold stratification to break dormancy [7]. *A. barbatum* seeds successfully germinated after receiving a 100 mg/L GA_3_ treatment (Fig. 4), proving that GA_3_ is a viable replacement for cold temperature. Moreover, the extended duration of the cold stratification period, lasting 3 months (Fig. 2), suggests the presence of intermediate PD instead of nondeep PD. Dormancy break and germination have been previously suggested to be more affected by the GA/ABA ratio than the overall GA content [30]. Hence, GA_3_ treatment induces dormancy breaking in *A. barbatum* seeds by increasing the GA/ABA ratio. As a result, we concluded that the seeds of *A. barbatum* exhibited intermediate complex MPD.

In other species of *Aconitum*, seeds of *A. heterophyllum* [35, 36], and *A. napellus* subsp. *castellanum* [37] were also reported to have intermediate complex MPD. By contrast, seeds of *Aconitum grossedentatum*, *A. lycoctonum* [38, 39], and *A. sinomontanum* [40] were reported to have deep complex MPD, which require cold temperature for an extended period due to loss of embryo PD and MD but GA_3_ did not promote germination [7]. Seeds of *A. altaicum*, *A. anthoroideum*, *A. lasiostomum* [19] were reported to have deep simple MPD, requiring warm temperatures followed by cold temperatures prior to germination. Interestingly, in her reference book on dormant seed germination, Nikolaeva (Russian) [19] reported that seeds of *A. barbatum* [19] had nondeep simple MPD. However, she did not specify how the dormancy of *A. barbatum* was broken. Such variations in dormancy types may be related to the different selection of habitats, which have caused the species to adapt ecologically and physiologically [39].

The dormancy mechanism observed in *A. barbatum* seeds may have evolved as an adaptation to the environmental conditions of its natural habitat. Seeds of *A. barbatum* are dispersed in the early fall. At seed dispersal, a small portion of seeds exhibit morphological dormancy and a large portion of seeds exhibit intermediate complex MPD (the combination of MD with PD). The delay in seed germination during the fall season, when temperatures are dropping, ensures that seedlings do not emerge until the following spring when conditions are more favorable for their survival. Dormancy breaking can be achieved in the field in winter if the daily minimal temperature is suitable for cold stratification. Such mechanism allows *A. barbatum* to avoid unfavorable winter temperatures and potential damage from frost or snow. Furthermore, seeds of *A. barbatum* had increased germination percentages in light compared with under darkness, indicating that light promote germination. Seeds on soil surface will thus possibly exhibit higher germination percentages. Many herbaceous plants benefit from light for seed germination [31–33]. Given the unpredictability of the environment, the seed delay mechanism may be most likely an ecologically advantageous strategy [7, 31, 32, 34]. As a result, the dormancy mechanism in *A. barbatum* seeds likely represents an evolutionary strategy for successful reproduction and propagation in its environment.

## Conclusions

During seed dispersal, *A. barbatum* seeds are in the dormancy with undeveloped embryos. Under natural environmental circumstances, embryo development begins in early winter. The E/S ratio increases to from 0.33 to 0.78 before the radical emerges. Laboratory experiments showed that embryo growth was observed following long-time incubation under 4 °C. Seeds were able to germinate over a broad temperature range following cold stratification under 4 °C, and GA_3_ may be used as an alternative to cold stratification. We conclude that fresh seeds of *A. barbatum* display intermediate complex morphophysiological dormancy. Natural cold stratification in winter breaks dormancy, allowing seeds to germinate and sprout seedlings at the beginning of following growing season. Those findings are useful to horticulturists and seed ecologists in analyzing *A. barbatum* germination and promoting seed propagation.

## Materials and methods

### Seed collection

In late September of 2018, the newly ripened *A. barbatum* seeds in several hundreds of naturally growing plants in Xinbin County, Liaoning’s Fushun, China (41°56′N, 125°4′E) were collected. We dried the seeds for 5 days (at ambient room conditions; 20–25 °C with 40–50% RH) before conducting the germination experiment. Note that this species is widely distributed in the natural forests and grasslands of northern China. Therefore, collection permission is not required, and a voucher specimen of the plant materials does not need to be deposited in a publicly available herbarium. The experimental research conducted on plants in this study adhered to institutional, national, and international guidelines.

### Seed mass and water content

We weighed ten replicates for 100 randomized seeds using the electronic scale with an accuracy of 0.0001 g. Seed length, width and thickness of ten seeds were determined by the vernier calipers. The fresh seed water content was determined as follows: weight of ten duplicates of 100 seeds was determined with the analytical scale to a constant weight before and after 48 h of drying at 80 °C in an oven. The seed water content was determined as the ratio of the difference between the fresh weight and dry weight to the fresh weight.

### Imbibition tests

Seed water absorption was analyzed in the laboratory to measure seed coat permeability at 20–25 °C under 40–50% RH. Each replicate involved 25 seeds that were duplicated four times. The seed weight was measured before being placed in a plastic Petri dish containing Whatman No. 1 filter paper soaked in distilled water. At 0, 1, 3, 6, 9, 12, 24, and 48 h of water absorption, the seeds in the Petri plates were removed, dried, weighed, and replaced into each dish. Percent increase of fresh mass was determined below: %*W*_r_ = [(*W*_f_–*W*_i_)/*Wi*]×100, where *W*_r_, *W*_i_, and *W*_f_ are percent increase in mass, original seed mass as well as mass following a specific period, respectively [12].

### General experimental procedures for laboratory germination

Four replicates were used in the entire laboratory germination tests, each involving 25 seeds per treatment. After being dispensed onto the dual-layer filter paper, distilled water (5 mL) was used to moisten the seeds on a plastic Petri dish (diameter: 10 cm). To prevent intra-incubation loss of water, parafilm was utilized to seal every Petri dish and its lid. Whenever necessary, water was replenished into the dishes to maintain the moistness of the filter paper. For the germination trails, seed incubation was carried out in 12-h/12-h light/dark photocycles or persistent dark (by placing the dishes into black bags) conditions at 5/15, 10/20, 15/25 and 20/30°C. Those temperatures stimulated the average daily minimal/maximal air temperatures during the whole growing season at *A. barbatum* seed gathering site, and across eastern China, i.e., 5/15°C in Mar., early Apr. and Nov.; 10/20°C from mid-late Apr. to Oct.; 15/25°C from May to mid-late Sept.; and 20/30°C from June to early Sept. The minimal and maximal temperatures separately coincided with the diurnal 12-h light/12-h dark photocycle for seeds, which were subjected to incubation under ca. 100 µmol/m^2^/s photon irradiance (400–700 nm) of light offered by cool white fluorescent tubes. The germination criterion was ≥ 1 mm radicle tip emergence [27]. After completion of the experiments, any ungerminated seeds were pinched with forceps to identify whether a viable firm white or non-viable soft gray embryo was present [7].

### Germination and embryo length of fresh seeds

In this experiment, seeds of *A. barbatum* seeds were incubated in darkness or light under 5/15, 10/20, 15/25 and 20/30°C for 30 days. The germination process was monitored on a daily basis. In addition, ten seeds with four duplicates were immersed for a 24-h period within Petri dishes with water-moistened filter paper in the laboratory. The embryo and seed lengths were measured by dissecting microscope. The ratio of embryo-to-seed length (E/S) was determined to analyze embryo growth.

### Effects of cold/warm stratification on germination of *A. barbatum* seeds

Fresh *A. barbatum* seeds were placed in metal boxes (10 cm deep, 20 cm diameter) between two filter paper layers onto the rinsed quartz sand (water content of 11–14%) and incubated at 4 or 25 °C for determining seed responses to cold/warm stratification. After 4, 8, 12, and 16 weeks, four duplicates of 25 ungerminated seeds were randomly selected from each box and transferred to Petri dishes for a 30-day incubation in light and dark at 5/15, 10/20, 15/25, or 20/30°C. Every day, the germinated seed amount was determined daily.

### Role of temperature in *A. barbatum* seed embryo development

Seven Petri dish each containing ten seeds were incubated in the light for 14 weeks at 4 or 15/25°C to explore how temperature affected embryo development. At 2-week intervals, we selected one Petri dish under every temperature for determining E/S ratio for 10 seeds.

### Effects of GA_3_ on germination of *A. barbatum* seeds

This experiment involved four duplicates of 25 freshly collected *A. barbatum* seeds cultivated in the light for 12 weeks at 5/15, 10/20, 15/25, and 20/30°C using 5 mL of GA_3_ solution (0, 10, 100, 1,000 mg/L). Filter papers were regularly moistened with distilled water. The germinated seed amount was determined every week and discarded.

### Germination phenology and embryo growth

On October 1, 2018, 60 nylon bags containing 25 seeds together with sands (10 g) were buried into the soil at Yangzhou University’s experimental garden in to investigate the embryo development and seed germination phenology of *A. barbatum* seeds in the field. After being buried into two plots (1 m^2^), nylon bag locations (1 mm mesh size) were recorded. To keep birds outside, each plot was enclosed with a net. Every two weeks, four bags were dug up to count the number of seeds that germinated. Under a microscope, ten seeds in every bag were utilized for measuring embryo/seed lengths. After removing any seeds with radicle protrusions from embryo analysis, we determined E/S ratio. A digital thermometer was used to measure the daily air temperatures. Given that our seeds were collected in northern China and the field experiments were conducted in eastern China, it is important to note that there could be potential differences in the germination characteristics of the seeds compared to those germinated in their natural habitat. However, our main objective was to identify the dormancy types exhibited by *A. barbatum* seeds. Dormancy types are formed as a result of the long-term process of evolution and adaptation [7], and it is unlikely that these traits would change significantly during a short period of six months of incubation under controlled conditions.

### Data analyses

We converted the data concerning seed germination to the percentage of alive seeds. Before analysis, arcsine transformation of the percentage data was accomplished for ensuring homogeneity of variance. We utilized two-way ANOVA to analyze how light, temperature together with the combination affected seed germination, how temperature regimes and incubation period affected embryo development, and how GA_3_ dose, incubation temperature, as well as their combination affected germination. Moreover, we applied three-way ANOVA for assessing how light, stratification, incubation period along with the combination affected seed germination. In case statistical significance of a difference was confirmed by ANOVA, while Tukey’s HSD test was employed to ascertain the inter-treatment differences (P < 0.05). SPSS ver. 20.0 (SPSS, Chicago, IL, USA) was applied for the entire data analyses.

## Data Availability

The data generated or analyzed in this study are included in this article. Other materials that support the findings of this study are available from the corresponding author on reasonable request.
